# Unraveling implementation context: the Basel Approach for coNtextual ANAlysis (BANANA) in implementation science and its application in the SMILe project

**DOI:** 10.1186/s43058-022-00354-7

**Published:** 2022-10-01

**Authors:** Juliane Mielke, Lynn Leppla, Sabine Valenta, Leah L. Zullig, Franziska Zúñiga, Sandra Staudacher, Alexandra Teynor, Sabina De Geest

**Affiliations:** 1 Institute of Nursing Science (INS), Department Public Health (DPH), Faculty of Medicine, University of Basel, Bernoullistrasse 28, CH-4056 Basel, Switzerland; 2 Department of Medicine I, Faculty of Medicine, Medical Center University of Freiburg, Freiburg im Breisgau, Germany; 3 Department of Hematology, University Hospital Basel, Basel, Switzerland; 4 Center for Innovation to Accelerate Discovery and Practice Transformation (ADAPT), Durham Veterans Affairs Health Care & System, and Department of Population Health Sciences, School of Medicine, Duke University, Durham, NC USA; 5 Department of Health Services Research, Care and Public Health Research Institute, Maastricht University, Maastricht, The Netherlands; 6 University of Applied Sciences Augsburg, Faculty of Computer Science, Augsburg, Germany; 7 Academic Center for Nursing and Midwifery, Department of Public Health and Primary Care, KU Leuven, Leuven, Belgium

**Keywords:** Context, Contextual analysis, Implementation science, Methodology

## Abstract

**Background:**

Designing intervention and implementation strategies with careful consideration of context is essential for successful implementation science projects. Although the importance of context has been emphasized and methodology for its analysis is emerging, researchers have little guidance on how to plan, perform, and report contextual analysis. Therefore, our aim was to describe the Basel Approach for coNtextual ANAlysis (BANANA) and to demonstrate its application on an ongoing multi-site, multiphase implementation science project to develop/adapt, implement, and evaluate an integrated care model in allogeneic SteM cell transplantatIon facILitated by eHealth (the SMILe project).

**Methods:**

BANANA builds on guidance for assessing context by Stange and Glasgow (Contextual factors: the importance of considering and reporting on context in research on the patient-centered medical home, 2013). Based on a literature review, BANANA was developed in ten discussion sessions with implementation science experts and a medical anthropologist to guide the SMILe project’s contextual analysis. BANANA’s theoretical basis is the Context and Implementation of Complex Interventions (CICI) framework. Working from an ecological perspective, CICI acknowledges contextual dynamics and distinguishes between context and setting (the implementation’s physical location).

**Results:**

BANANA entails six components: (1) choose a theory, model, or framework (TMF) to guide the contextual analysis; (2) use empirical evidence derived from primary and/or secondary data to identify relevant contextual factors; (3) involve stakeholders throughout contextual analysis; (4) choose a study design to assess context; (5) determine contextual factors’ relevance to implementation strategies/outcomes and intervention co-design; and (6) report findings of contextual analysis following appropriate reporting guidelines. Partly run simultaneously, the first three components form a basis both for the identification of relevant contextual factors and for the next components of the BANANA approach.

**Discussion:**

Understanding of context is indispensable for a successful implementation science project. BANANA provides much-needed methodological guidance for contextual analysis. In subsequent phases, it helps researchers apply the results to intervention development/adaption and choices of contextually tailored implementation strategies. For future implementation science projects, BANANA’s principles will guide researchers first to gather relevant information on their target context, then to inform all subsequent phases of their implementation science project to strengthen every part of their work and fulfill their implementation goals.

**Supplementary Information:**

The online version contains supplementary material available at 10.1186/s43058-022-00354-7.

Contributions to the literature

- We provide a comprehensive, multi-component approach to guide contextual analysis in implementation science, i.e., the Basel Approach for coNtextual ANAlysis (BANANA).
- BANANA specifically provides guidance on how to combine theories, models, and frameworks; how to use empirical evidence in contextual analysis; how to choose a study design for assessing context and setting; how to use findings from the contextual analysis to inform subsequent phases of an implementation science project (e.g., intervention development/adaption, implementation strategies); and how to report contextual analyses.
- Using a case example, we demonstrate a successful application of BANANA in an ongoing implementation science research project.

## Background

The importance of context^1^ for a successful and sustainable implementation has gained significant attention in implementation science (IS) with contextual analysis increasingly being recognized as vital to IS methodology [1–4]. While contextual analyses’ value is widely accepted, guidance on how to conduct one is lacking and no unified definition of contextual analysis in IS exists. We understand contextual analysis as a foundational phase within IS projects to which specific research questions and IS theories, models, or frameworks (TMFs) are applied [3, 5, 6]. It entails the mapping of relevant qualitative and quantitative information about the context (e.g., multilevel implementation determinants, practice patterns) in which an intervention will be delivered. Starting (prospectively) at the beginning of each IS project, the results of the contextual analysis become the basis of all subsequent phases of an IS project: they inform intervention development or adaption, guide choices regarding implementation strategies, help interpret implementation and effectiveness outcomes, and guide selection of sustainability strategies [7–10]. As context is dynamic and evolves, continuous monitoring of context including for example several assessments of context throughout the project is important. Our view is that contextual analysis requires methodological strengthening as very limited guidance is available so far and conceptual and methodological unclarity on contextual analysis exists.

Conceptual inconsistencies between the applied methods and approaches hamper the development of a standardized approach [11]. In their systematic review of 64 empirical implementation studies, Rogers et al. identified over 40 distinct strategies to study context via quantitative, qualitative, and mixed-methods approaches [11]. Whereas assessment of contextual factors often focuses on the meso-level (e.g., organizational culture and climate, readiness for implementation), macro-level factors (e.g., political and economic climate) are rarely considered [12–16].

TMFs provide guidance on which contextual factors to study, but not on how to study context per se [17–19]. Commonly applied TMFs that incorporate context include the Consolidated Framework for Implementation Research (CFIR) [20], the Integrated Promoting Action on Research Implementation in Health Services (i-PARIHS) framework [21], or the Theoretical Domains Framework (TDF) [22]. This emphasis on theory contrasts with an increasing number of IS studies that focus on mapping single facilitators and barriers to implementation, but that follow no specific theory. This absence both obscures the researchers’ rationale for choosing their variables and limits theoretical development based on empirical evidence. Along with any multilevel perspective, they also commonly lack assessments of interactions between context, intervention, and implementation [11, 15, 23–27]. And what contextual information they generate is rarely linked to subsequent phases in their IS project [28]. In cases where contextual analysis is treated not as a separate foundational phase of an IS project, but as an add-on, contextual data are commonly only performed retrospectively, as part of a process evaluation [19, 29]. This obviously excludes any chance of applying any contextual information to the IS project’s next phases. Furthermore, as findings of contextual analyses are rarely published (particularly meso-level factors, e.g., in regard to practice patterns), transparency of a project might be limited as valuable methodological observations, e.g., in regard to intervention development, that might be important to other researchers’ projects are not reported ([30]; Mielke J, Brunkert T, Zúñiga F, Simon M, Zullig L, De Geest S: Methodological approaches to study context in implementation science studies: an evidence gap map, Under review). To conclude, a comprehensive guidance focusing not only on individual aspects of a contextual analysis (e.g., theoretical underpinning or methods to assess context) is lacking in implementation science.

As part of a series of guidelines commissioned by the Agency for Healthcare Research and Quality, Stange and Glasgow [31] provided an initial step-wise approach to assessing and reporting contextual factors throughout the phases of patient-centered medical home research. Since this approach was not initially developed for IS projects, it lacks further details on the operationalization of context, specific methods to assess context, and the use of contextual information to inform later IS project phases. To fill these gaps and to provide guidance “how to do” contextual analysis in IS projects, we developed the Basel Approach for coNtextual ANAlysis (BANANA), which entails six components. Accordingly, this paper has two objectives: first, to describe the six components of BANANA; and second, to describe its application to the SMILe project, i.e., development/adaption, implementation, and evaluation of an integrated care model (ICM) in allogeneic stem cell transplantation facilitated by eHealth (SMILe) (Table 1) [32, 33].

**Table 1:** Description case example SMILe project^a^ ([32–34]; Valenta S, Ribaut J, Leppla L, Mielke J, Teynor A, Koehly K, Gerull S, Grossmann F, Witzig-Brändli V, De Geest S, for the SMILe study team: Context-specific adaptation of an eHealth-facilitated, integrated care model and tailoring its implementation strategies – a mixed-methods study as a part of the SMILe implementation science project, Under review)

**Background**	
Follow-up care of allogeneic hematopoietic stem cell transplanted (alloSCT) patients is challenged due to growing numbers of alloSCT transplant survivors who have complex care needs. Current follow-up models are biomedically driven rather than focusing on behavioral, psychosocial, and self-management support elements.	
**Aim SMILe project**	
SMILe is an implementation science project to develop/adapt, test, and implement an eHealth-facilitated integrated care model (ICM) in allogeneic stem cell transplantation (SMILe-ICM).	
**Methods**	
SMILe is a multi-site project consisting of phase A (contextual and technology acceptance analysis and development of intervention and implementation strategies) and phase B (implementation and testing of the intervention). Phase A has been completed in two transplant centers in Germany (intervention development) and Switzerland (intervention adaptation), and further centers in Belgium and Switzerland will follow. The SMILe-ICM is currently being implemented and tested (phase B) using a hybrid type 1 effectiveness-implementation design at two study sites in Germany and Switzerland, and first results are expected in 2022 and 2023.	
**Intervention**	
The SMILe-ICM is based on the eHealth enhanced Chronic Care Model and targets patients, healthcare providers, and the system [35]. It includes a human and an eHealth component. The human component is an advanced practice nurse (SMILe Care Coordinator), who provides self-management support and health behavior promotion via face-to-face visits [33, 36]. The SMILe App (eHealth component) allows the patient to enter values on a daily basis and send them to the transplant center. Via SMILeCare, the Smile Care Coordinator can overview incoming data regularly, allowing early detection of health deterioration. Face-to-face visits can be adapted according to patients’ needs.	
**Contextual analysis — aims**	
The aims of the contextual analysis were as follows: (1) to identify the target organization’s structural characteristics and practice patterns in view of chronic illness management, (2) to assess how self-management and behavioral support are currently supported, (3) to assess the technology openness of clinicians and alloSCT patients regarding eHealth use along the eCCM dimensions, and (4) to explore facilitators and barriers to SMILe-ICM implementation (only assessed in second study site to date).	

^a^SMILe project: development/adaption, implementation, and evaluation of an integrated care model (ICM) in allogeneic stem cell transplantation facilitated by eHealth (SMILe)

## Methods

To develop BANANA, we used a multiphase approach. First, we conducted a literature review, focusing on methodological IS papers available via major electronic data bases (PubMed, EMBASE, and Web of Science). To identify existing methodological approaches for contextual analysis, we screened the identified papers’ reference lists. The only authors to provide a methodological description of an entire contextual analysis were Stange and Glasgow [32]; others addressed only individual aspects, e.g., the use of TMFs or methods to study context [3, 11, 31]. However, as Stange and Glasgow’s approach is not IS specific, we used it as a basis to develop BANANA and adapted it as necessary to guide a contextual analysis for the SMILe project and for IS projects in general. Briefly, SMILe is a multi-site, multiphase IS project (Table 1): Phase A entailed analyzing the context and target users’ technology acceptance, as well as developing/adapting and extending the SMILe-ICM and its setting-specific implementation strategies [34, 36, 37]. In fact, BANANA was originally developed to guide contextual analysis in this phase to inform intervention development (German setting) [34] and intervention adaptation (Swiss setting). Phase B focused on the SMILe-ICM’s implementation and evaluation using a hybrid effectiveness-implementation randomized controlled trial [32, 33].

Second, research group members (SDG, LL, SV, AT, JM) consulted with IS experts (LLZ, FZ) and a medical anthropologist (SS) in iterative discussion sessions about the identified literature and how to elaborate Stange and Glasgow’s approach in view of its application for IS projects. We used the three-step approach as a basis, adapted it to IS (e.g., IS terminology, relevance of context for different phases of an IS project), and complemented it with identified evidence on context and its assessment (e.g., methods for data collection) as well as other relevant aspects (e.g., use of empirical evidence) (Additional file 1). Our understanding of context and how it is reflected in BANANA was conceptually based on the Context and Implementation of Complex Interventions (CICI) framework [1] and is described elsewhere in detail [38]. CICI is a meta-framework that in contrast to other frameworks explicitly focuses on the multilevel, dynamic context, i.e., interactions between intervention, implementation, and context. CICI operationalizes context across seven domains (geographic, epidemiological, socio-cultural, socio-economic, political, legal-ethical). Each of these entails micro-, meso-, and macro-level contextual factors. CICI differentiates between setting and context, defining it as the physical location in a context in which an intervention is implemented [1]. In the setting interactions between the intervention, the implementation and the other contextual factors occur. Thus, the contextual analysis includes an assessment not only of contextual aspects but also of the setting in which the implementation takes place. BANANA spans around the CICI’s constructs (i.e., multidimensional, multilevel, dynamic) and acknowledges in its methodological guidance differences between context and setting. Starting with an initial version of BANANA compiled by the first and last authors (JM, SDG), we further refined each of the steps (e.g., specifying reporting of contextual findings). After ten discussion rounds between all study authors, we reached a consensus on BANANA (i.e., conceptual underpinning, different components, and methodological aspects). The initial and final version of BANANA, as well as which aspects of BANANA were informed by the CICI framework, can be found in Additional file 1.

## Results

The BANANA approach entails six components (Table 2): (1) choosing a TMF, (2) using empirical evidence, (3) involving stakeholders, (4) designing a study specifically for the contextual analysis, (5) determining the relevance of contextual factors for implementation strategies/outcomes and intervention co-design, and (6) reporting on the contextual analysis. Stakeholder involvement represents a key element of contextual analysis and thus relates to all other components. BANANA’s components are presented linearly, but depending on the project, components 1–3 can occur concurrently and determine the need and focus for component 4. Meaning that depending on the project aims and context information available, researchers need to carefully review in their specific case whether component 4 of BANANA is relevant to gain further contextual information and to reflect on how to realize those components. Working in an interdisciplinary research team that combines different expertise and skills can therefore be instrumental in planning and executing a contextual analysis. BANANA is explained in detail below; for each component, an example from the SMILe project is provided. We also provide further key resources in regard to the different components of BANANA in Additional file 2 that provide further guidance. A detailed description of the SMILe study and its methods for contextual analysis can be found elsewhere [34].

**Table 2:** Description of the six components of the Basel Approach for coNtextual ANAlysis (based on Stange and Glasgow [31])

**Component 1**	**Choose a theory, model, or framework (TMF) to guide contextual analysis**
	Considerations when selecting a TMF for contextual analysis – TMF acknowledges the multidimensional, multilevel, and dynamic nature of context – TMF fits the intervention and/or setting in which the intervention will be implemented *Consider combination of a context and setting-specific TMF*
**Component 2**	**Use empirical evidence to identify relevant contextual and setting factors**
	Identification of empirical evidence on relevant contextual and setting factors for implementation using various sources of evidence [39] – Local data and information – Professional knowledge/clinical experience – Patient experiences and preferences – Research
**Component 3**	**Involve stakeholder**^**a**^
	– Identification and listing of relevant stakeholders for contextual analysis (target group, implementers, decision-makers, others) from different levels (micro, meso, macro) – Mapping of stakeholders in a stakeholder matrix specifying their characteristics (e.g., influence, role, activity, product) – Visualizing stakeholder characteristics in an influence-interest-capacity matrix – Verifying stakeholder availability and commitment – Developing a stakeholder strategy specifying stakeholder tasks, timepoints, and methods for involvement – Evaluation of stakeholder involvement and adaption if needed
**Component 4**	**Develop a study design for contextual analysis**
	Data collection is guided by theory, empirical evidence, and stakeholder input Choice of appropriate methods to answer the research questions such as – Quantitative methods (e.g., survey, routine data) – Qualitative methods (e.g., interview, focus group, observation) – (Rapid) ethnography *Consider changes of context over time* *Plan (if possible) several timepoints for data collection (e.g., prior, during, and at the end of the project)*
**Component 5**	**Determine the relevance of context for intervention co-design, choice of implementation strategies, and interpretation of outcomes**
	Findings from the contextual analysis can be used for: – Development/adaption of the intervention – Choice/adaption of implementation strategies – Interpretation of implementation and effectiveness outcomes – Choice of sustainability strategies *Consider the development of a program theory to describe/visualize causal pathways between intervention components, implementation strategies, and contextual factors*
**Component 6**	**Report on contextual analysis**
	Reporting contextual analysis as part of the implementation intervention study (detailed findings can be reported in a separate paper) Suggestions for reporting based on BANANA: – Definition of context and operationalizations of contextual and setting factors studied – TMF applied for contextual and setting analysis and description of how it was used – Overview of empirical evidence identified and used – Stakeholder involvement (i.e., stakeholder strategy) – Reporting methods applied for data collection and analysis (e.g., study design, measures used, contextual and setting factors assessed) – Use of findings from the contextual analysis for subsequent project phases (cf. component 5)

^a^Adapted from Barkhordarian et al. [40]

### Component 1: Choosing a TMF to guide contextual analysis

#### Identification and selection of TMFs

In general, the use of TMFs is essential to inform and guide all phases of IS projects and increase the findings’ generalizability [24, 41–43]. Regarding contextual analysis, a TMF can serve as “a comprehensive starting point” to identify contextual factors that influence implementation.

The selection of a framework is often perceived as difficult, as a large number of IS and other TMFs are available [24, 41, 44, 45]. Therefore, following Moullin et al.’s recommendations [46], we suggest considering four criteria when selecting a TMF: (1) it is intended/designed for contextual analysis; (2) it acknowledges the multidimensional, multilevel, and dynamic nature of context; (3) it includes guidance on operationalization of concepts (e.g., contextual factors); (4) it fits the intervention and setting.

Resources that provide an overview of TMFs or support the identification, selection, and combining of TMFs include key IS papers [3, 24, 44, 47] and tools such as the D&I Models Webtool [48]. To justify and report TMF selection, the implementation theory comparison and selection tool (T-CaST) can provide useful guidance [41]. Based on 16 criteria relating to applicability, usability, testability, and acceptability, T-CaST provides a first attempt to select and compare TMFs [41]. Furthermore, to ensure a TMF’s fit and applicability for a specific setting and/or context, stakeholders can be involved (cf. component 3) [46].

#### Combining of TMFs for context and setting

As context differs from the setting^1^, we suggest the combination of a context- and setting-specific TMF, as such combinations enhance the granularity of contextual analysis. While context-specific TMFs provide an overview of factors that may influence implementation (e.g., socio-cultural characteristics), setting-specific TMFs indicate factors that influence a specific intervention’s implementation in a specific setting (e.g., site characteristics, practice patterns, work flows, and processes within that setting). A broad variety of TMFs are available for specific settings and/or interventions, e.g., the Chronic Care Model [49] or the Primary Care Practice Development Model [39].

#### Case example — use of TMFs in the SMILe project

In the SMILe project, we chose the CICI framework [1] as an overarching framework for contextual analysis. In our view, as it acknowledges contextual dynamics and distinguishes between context and setting, it is currently the most mature framework available. Working with the CICI framework, we assessed relevant micro- and meso-level contextual factors from the three context domains—geographic (i.e., Internet access, type of connection), epidemiological (i.e., patient demographics), and socio-cultural (i.e., self-management, health behavior). We did not explicitly assess further contextual factors. As SMILe project leaders (LL, SV) themselves have been working for years within the SMILe-ICM’s implementation setting, they had implicit and explicit contextual knowledge (e.g., organizational culture, leadership, and legal aspects).

**Fig. 1:**
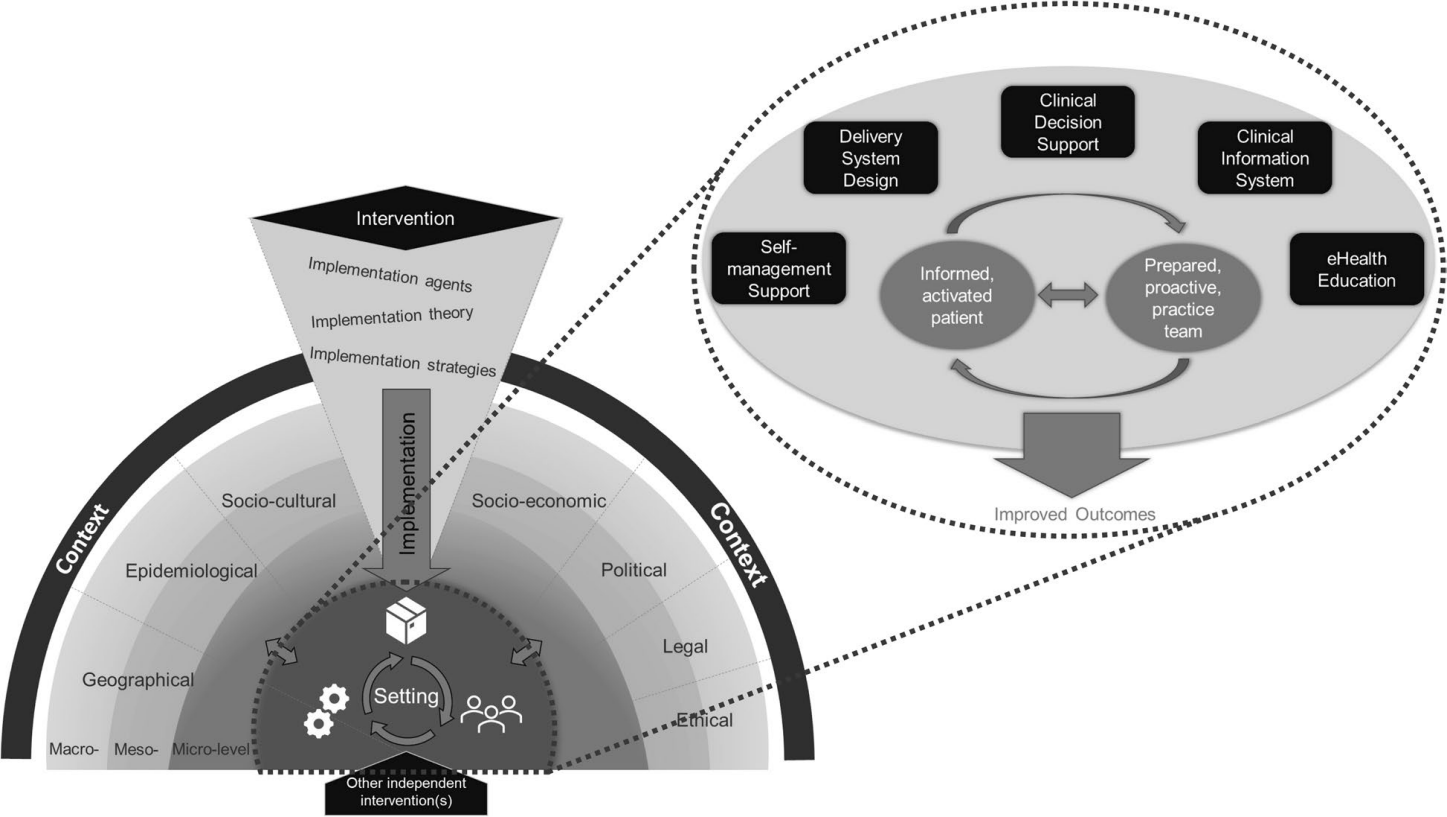
Combination of the Context and Implementation of Complex Interventions (CICI) framework [1] and the eHealth Enhanced Chronic Care Model (eCCM) [35] to guide contextual analysis within the SMILe project. Figure adapted from Pfadenhauer et al. [1] and Gee et al. [35]

As the SMILe project’s focus is on developing and implementing an eHealth-facilitated ICM, we combined the CICI framework with the eHealth Enhanced Chronic Care Model (eCCM) to gain a deeper understanding of the target setting (the stem cell transplant center) (Fig. 1) [1, 35]. The eCCM supports the re-design of acute care-oriented processes towards chronic illness management [35, 49]. The SMILe researchers assessed factors from the model’s five building blocks (i.e., self-management support, delivery system design, clinical decision support, clinical information systems, eHealth education) [34]. Micro-level factors of interest included self-management support and technology openness; on the meso-level, they included transplant center structural characteristics, practice patterns in follow-up care, the level of chronic illness management, team composition, and clinician demographics [35]. Macro-level factors were considered but not explicitly assessed and reported (e.g., legal aspects).

#### Component 2: Using empirical evidence for contextual analysis

TMFs provide a comprehensive overview of how context is conceptualized and which contextual factors are relevant for implementation. However, not all aspects mentioned in the TMFs are relevant to each IS project. Therefore, using available empirical evidence can help to determine what is already known about the specific implementation context and relevant contextual factors. Four sources of evidence exist, i.e., (1) local data and information, (2) professional knowledge/clinical experience, (3) patient experiences and preferences, and (4) research [50]. The first three can be considered through stakeholder input (cf. component 3); local data can be also identified, e.g., by studying audit and performance data. To assess evidence from research, a literature review on relevant contextual factors should be conducted [1, 11, 51].

Additional file 3 provides an overview of micro-, meso-, and macro-level contextual factors we identified (via our literature review) as the most commonly reported influencing implementation [11, 13, 27, 52]. Additionally, Rogers et al. identified team-level factors (e.g., team characteristics and teamwork, team stability, morale) important to implementation [11]. Reviews such as these provide broad views of relevant contextual factors. However, whenever possible, researchers need to consider evidence on implementation determinants for specific interventions, target groups, or settings, e.g., Evans et al.’s research toolkit to study organizational contextual factors influencing the implementation of ICMs [53]. That toolkit includes a framework and measurement tools to study those factors.

Empirical evidence can shed light on gaps in our understanding of context and can point to aspects of context that need further inquiry. It thus informs the focus and extent of the primary data collection in component 4 or even unravels in certain circumstances that no further primary data collection is required to inform further steps of the implementation project [1].

#### Case example — use of empirical evidence in the SMILe project

In order to optimally inform the SMILe contextual analysis, all sources of evidence were used. First, a literature review revealed limited evidence on follow-up practice patterns in allogeneic stem cell transplantation (research evidence). Other identified studies reported on challenges with eHealth implementation (e.g., low adoption rates) [54–57], including relevant contextual factors that tend to hinder implementation (e.g., technology acceptance, interoperability of technology, financial resources) [58–62]. The findings of the literature review served as the basis for deciding which factors to explore in more detail as part of our contextual analysis [63]. Based on the factors identified, questionnaire surveys and interview guides for contextual analysis were chosen—and, if necessary, complemented—to clarify our picture of the studied context. For example, as part of the contextual analysis, target patients’ technology openness was assessed and patients’ and clinicians’ experiences using eHealth to support health or healthy behaviors explored [34, 64].

Second, in addition to the literature review, our studies in allogeneic stem cell transplantation as well as clinical experience of the SMILe team and patient feedback highlighted the challenges patients face in trying to improve their self-management behavior, e.g., medication non-adherence or physical activity [63, 65]. Based on this evidence, we added specific questions about self-management challenges and how to overcome them to our interviews and focus groups [34].

Furthermore, the SMILe project leader’s knowledge among others about the organizational culture, leadership, work processes, and structures in the implementation setting, helped (1) to specify factors relevant to consider in the contextual analysis (e.g., level of chronic illness management); (2) to identify relevant stakeholders in the clinical setting and to rate their influence on the implementation; (3) to provide a basic understanding of the setting’s readiness for change and their openness towards eHealth technology; and (4) to inform the study design (e.g., feasibility of recruitment strategy and inclusion/exclusion criteria) and selection of appropriate methods (e.g., a combination of individual and focus group interviews with clinicians was selected due to conflicting time schedules). Finally, information on the setting’s resources (e.g., financial, staffing) and operability of the IT system were gained in individual, informal stakeholder meetings (local data and information). Those meetings were essential for the context-based development and adaptation of the SMILe-ICM, to identify potential hindering factors for its implementation in the specific setting and plan ahead for its sustainability.

### Component 3: Stakeholder involvement in contextual analysis

Stakeholder involvement is essential in every component of a contextual analysis. Stakeholders are “those individuals [or organizations] without whose support and feedback an organization, or a project within an organization [or beyond] cannot subsist” [40]. They can be targeted or affected by an intervention (e.g., patients and caregivers), actively implementing an intervention (e.g., healthcare practitioners), deciding on whether it will be implemented (e.g., organizational leaders, policy makers) [1, 66–69]. It is also possible to ask input from specific experts (e.g., epidemiologists, researchers) on dedicated topics.

#### Identification of stakeholders and development of a stakeholder strategy

The matter of which stakeholders to involve in contextual analysis always depends on the project’s specific focus [40]. To help ensure productive and robust stakeholder involvement, developing a stakeholder strategy is key. This indicates which and how stakeholders will be involved at each step of the contextual analysis, specifies each group’s tasks, and outlines methods or tools to involve each group. Essentially, stakeholder selection must be systematic, i.e., involving multiple stakeholder perspectives from every relevant level (micro, meso, and/or macro), while balancing power and bridging inter-group disparities, e.g., between patients and care specialists. Identified stakeholders can be mapped on a matrix (i.e., influence-interest-capacity matrix) that specifies their characteristics, e.g., role, degree of influence, anticipated effects, and outcomes of involving them [40]. Throughout the project, continuous changes in context require continuous involvement of the stakeholders, e.g., via regular stakeholder meetings [10, 30]. Furthermore, their needs must be continuously evaluated and adapted as necessary.

#### Stakeholder tasks and tools for involvement

Since no specific guidance is available regarding stakeholder involvement in IS projects, general guidelines such as INVOLVE [70] or the PARADIGM Patient Engagement Toolbox [71] can support researchers to plan stakeholder tasks and choose tailored tools for stakeholder involvement. Within a contextual analysis, stakeholder tasks can include helping to choose a TMF, identifying/selecting relevant contextual factors for analysis, and evaluating and monitoring those factors throughout the project. By helping research teams interpret the findings of the contextual analysis, stakeholders can also deepen their understanding of inherent inter-factor relationships. Further tasks include supporting the development of contextually adapted intervention and implementation strategies [31, 46, 66, 72]. In these ways, stakeholder involvement can contribute to interventions’ acceptance, adoption, and feasibility. That is, engaged stakeholders will add considerably to an intervention’s successful implementation and sustainment [73].

#### Case example — stakeholder involvement in the SMILe project

The SMILe project involves stakeholders at multiple levels throughout the project and thus also in the contextual analysis [34]. Potential stakeholders were identified in brainstorming sessions and via one-to-one in-depth discussion by the SMILe research team, project leaders, and clinicians working in the field. Selections were based on their expert opinions and their perceptions of the stakeholder’s influence in regard to the implementation process and sustainability of the SMILe-ICM as well as their interest in the SMILe-ICM. In the second center, a stakeholder matrix was developed, indicating each of the stakeholder’s impact/influence, and necessary resources for their involvement (Valenta S, Ribaut J, Leppla L, Mielke J, Teynor A, Koehly K, Gerull S, Grossmann F, Witzig-Brändli V, De Geest S, for the SMILe study team: Context-specific adaptation of an eHealth-facilitated, integrated care model and tailoring its implementation strategies – a mixed-methods study as a part of the SMILe implementation science project, Under review). The final stakeholder group included the target group (stem cell-transplanted patients) and implementers (transplant team members, e.g., in- and outpatient nurses, physicians, and psycho-oncologists). Furthermore, extending the “typical end-users,” we also involved decision-makers (e.g., transplant directors, nursing directors, and head nurses) and other stakeholders including hospital IT and medical controllers and patients’ family members. In addition to being tremendously useful in identifying the most appropriate participants for focus groups, they participated in data collection, supported interpretation of study findings, helped develop/adapt the SMILe-ICM, and helped choose/adapt implementation strategies [34]. The stakeholders were involved longitudinally (i.e., over the whole project period), to keep track to changes in the dynamic and evolving context. Over the course of the contextual analysis, other stakeholders, including experts in medical device regulation and health insurers, were involved via individual in-depth interviews.

### Component 4: Study design for contextual analysis

As a contextual analysis in our view is a foundational phase, and thus functions as a separate study within IS projects, it requires additional considerations regarding research methods and study design. Data collection concerning relevant contextual factors is informed by components 1–3 of BANANA, i.e., theory, empirical evidence, and stakeholder input. The choice of methods is driven by the research questions. In addition, considering that available resources (time, funding, personnel) for contextual analysis are usually constrained, researchers need to strike a “balance between speed and rigor” [74]. This balance will influence how extensively a contextual analysis can be carried out and which methods can be applied [75].

#### Methods and measurement tools to study context

To deepen the research team’s understanding of the context, a combination of quantitative and qualitative methods is typically used [11, 76, 77]. Where mixed-methods approaches are used [78–81], the overall focus can be on quantitative data, qualitative data, or any supported mix of the two [78, 82].

Quantitative methods include numerous types of surveys (e.g., online surveys, paper-pencil questionnaires, telephone surveys), systematic interviews, direct observations, or routine data. Quantitatively assessed contextual factors include, e.g., implementation climate, organizational culture and climate, available resources, and readiness for change. Several reviews provide overviews of current measurement tools and their psychometric properties [14, 15, 25, 83–88]. Furthermore—for instance, on the CFIR [89] and EPIS framework [90] project websites—measurement and data extraction tools are available to assess aspects of context mentioned in the frameworks. However, before applying any such measurement tools, research teams must ensure that they are appropriate for their intended use, produce psychometrically sound results, and will be used consistently over time to ensure the comparability of results [16].

To explore qualitative contextual factors, interviews (unstructured or semi-structured), focus groups, observations, or document analysis^2^ can be applied [91]. Qualitative methods are particularly suitable to identify stakeholders’ preferences and needs, values, beliefs, and attitudes and how these influence their behavior. Published recommendations for the use of qualitative methods in IS include a white paper by the National Cancer Institute [91–94]. Furthermore, for certain frameworks, such as the CFIR or CICI, interview guides have been developed to guide the exploration of context constructs [1, 89].

Some of these quantitative and qualitative approaches have been criticized for focusing only on specific levels (e.g., the meso/organizational level) or “only provid[ing] a cursory view of complex and dynamic contexts” [29]. However, alongside quantitative and/or qualitative methods, ethnographic methods can complement both these types of data, thereby facilitating in-depth insights in organizational and contextual processes that influence implementation. An ethnographic approach can help highlight interactions within the context that remain undetected by other methods, but that may have a substantial impact on the intended implementation [29]. Furthermore, details that may not be obvious to the interviewee (e.g., ritualized everyday actions, cultural and social norms) or differences between what is said and what is done can be identified via ethnographic methods [95–97].

Considering the limited resources available for contextual analysis, the current trend is increasingly toward rapid qualitative or rapid ethnographic approaches. For example, the Rapid Assessment Procedure-Informed Clinical Ethnography (RAPICE) method combines rapid assessment procedures with ethnography [74, 98]. Initial evidence suggests that rapid research methods can be as effective and rigorous as traditional approaches but more time- and cost-effective [98–100]. However, a research team planning on using these methods for the first time should be aware that applying them effectively and efficiently may require special training, multiple attempts, and methodological adaptions to fit their research setting [99–102].

#### Timepoints for data collection

BANANA focuses on the prospective assessment of context; however, as context is dynamic and evolves further, timepoints for considering context should be planned through the IS project. This does not mean starting over with component 1 of BANANA, but rather “keeping the thermometers in the system.” This can be achieved, for instance, through repeated assessments (e.g., surveys or use of routine data) or other methods (e.g., observations, site visits, document analysis) as well as through regular exchanges or informal conversations with stakeholders [38]. Currently, little guidance is available regarding which contextual factors to record at which timepoints and how frequently [31, 103]. Further insights may be gained from Ariadne Labs’ Atlas Initiative that aims to develop a data repository of contextual factors related to the implementation success of different interventions in different settings and at different timepoints of analysis (before implementation, 6 weeks after implementation, and monthly after that) [104, 105].

#### Case example — SMILe project data collection and analysis

For the SMILe contextual analysis, an explanatory mixed-methods (quantitative/qualitative) design was applied based on the research aims formulated [34]. Specific aims of this analysis are described in Table 1. Data collection and analysis were guided by the eCCM and the CICI framework. First, questionnaire surveys were conducted with each participating center’s patients, clinicians, and transplant director. These questionnaires allowed us to assess each center’s structural characteristics, practice patterns regarding chronic illness management, overall level of chronic illness management, current levels of self-management behavioral, and technology openness and acceptance [34]. We also gathered the demographic characteristics of patients and clinicians.

The questionnaires cover the eCCM’s five building blocks and have been applied by the research team to previous work in heart and solid organ transplantation [64, 106–109]. All questionnaires were adapted as appropriate to the allogeneic stem cell transplant setting. In some cases, we supplemented the questionnaires with further contextually relevant factors (e.g., patients’ acceptance of symptom monitoring and data sharing), based on aspects described in the context domain of the CICI framework ([35]; Valenta S, Ribaut J, Leppla L, Mielke J, Teynor A, Koehly K, Gerull S, Grossmann F, Witzig-Brändli V, De Geest S, for the SMILe study team: Context-specific adaptation of an eHealth-facilitated, integrated care model and tailoring its implementation strategies – a mixed-methods study as a part of the SMILe implementation science project, Under review).

Second, to support our understanding of the quantitative findings and allow a deeper understanding of further aspects relevant to the development/adaptation and implementation of the SMILe-ICM (e.g., patient’s performance of self-management tasks, patient’s and clinician’s barriers to technology use), we conducted focus groups with clinicians as well as focus groups and individual interviews with patients. In both cases, our interview guides followed the eCCM’s building blocks [34]. In the second center where we implemented an adapted version of the SMILe-ICM, we explored factors facilitating or hindering the SMILe-ICM’s implementation by means of focus group discussions ([35]; Valenta S, Ribaut J, Leppla L, Mielke J, Teynor A, Koehly K, Gerull S, Grossmann F, Witzig-Brändli V, De Geest S, for the SMILe study team: Context-specific adaptation of an eHealth-facilitated, integrated care model and tailoring its implementation strategies – a mixed-methods study as a part of the SMILe implementation science project, Under review). An overview of the variables assessed in the surveys and themes explored during the focus group and individual interviews can be found in Additional file 4.

Quantitative and qualitative data were collected over a 1-year period. Ongoing changes in context (e.g., changes in leadership) were noted by the SMILe project leaders and documented. The team also had a regular exchange with stakeholders via informal conversations and official stakeholder meetings. The data analysis followed three eCCM-guided steps: (1) analysis of quantitative (descriptive tables) and qualitative results (meta-maps), (2) mapping of quantitative and qualitative findings in a joint display, and (3) reflection on findings and their implications for intervention development and choices of implementation strategies (again in a joint display) [34].

### Component 5: Identifying and describing the relevance of contextual and setting factors for intervention co-design, implementation strategies, and outcomes

Implementation success and sustainment of an intervention and the implementation strategy depend heavily on how well they align to the target context [8, 110–114].

#### Intervention development and selection of implementation strategies

Numerous frameworks/guidelines help researchers develop interventions and select implementation strategies. One of these is the Medical Research Council (MRC) guidance for the development and evaluation of complex interventions in healthcare. Whereas previously context was mainly considered during process evaluation, i.e., retrospectively, the MRC guidance now recommends examining interactions between the intervention and context across all phases of intervention development, implementation, and evaluation^3^ [114].

Another guidance focusing on both intervention development and implementation strategies is Bartholomew, Parcel, and Kok’s “Intervention Mapping”—a five-step process, the foundation of which is a contextual analysis [115]. Other methods that can be applied to match implementation strategies to contextual factors are concept mapping, group model building, and conjoint analysis [116].

Furthermore, originally designed to facilitate implementation strategy choices, the CFIR–ERIC Implementation Strategy Matching Tool speeds the identification of implementation strategies available in the Expert Recommendations for Implementing Change (ERIC) compilation. The ERIC compilation’s strategies address specific constructs described in the CFIR framework [7]. Just as with implementation strategies, specific sustainability strategies can also be selected to ensure that a successfully implemented intervention remains in clinical practice.

#### Adaption of interventions and implementation strategies

Even where proven intervention or implementation strategies are available, adaptions are usually required to ensure their effectiveness in a new context [110, 111, 117]. However, before making changes, it is necessary to distinguish between core intervention components—which have to be implemented as they are to achieve a desired effect—and those adaptable to various contexts [111]. Building on the idea of Intervention Mapping, Implementation Mapping was developed for use with interventions that have already been developed and tested [25]. To ensure that an adaptation is transparent and reproducible, a description should be given of which contextual details necessitate it and how the proposed adaption addresses those details [118]. Another source for adapting interventions is the three-step ADAPT guidance [110]. When adapting an intervention, it is always necessary to record which intervention components or implementation strategies were adapted, in which ways, and why. Frameworks such as FRAME [118] and FRAME-IS [119] can support this process.

#### Interpretation of implementation and effectiveness outcomes

An intervention’s likely effects will vary across contexts and settings [120]. The findings of the contextual analysis help to understand mechanisms that influence the implementation process (i.e., what was implemented and how well), and how these mechanisms will likely influence the intended intervention’s effectiveness. Usually, this component is part of a process evaluation [120].

To describe how and why a specific intervention leads to its expected effects, as well as to trace causal pathways between intervention components, implementation strategies, and contextual factors, it will be necessary to develop a program theory [114, 121].

#### Case example — relevance of the contextual analysis for development/adaption of the SMILe-ICM and implementation strategies

Contextual analysis guided the development/adaption of the SMILe-ICM and the selection of implementation strategies. All quantitative and qualitative findings were synthesized in a joint display and the intervention’s implications summarized. Identified gaps both in self-management (e.g., symptom recognition) and in delivery system design (e.g., chronic care delivery, continuity of care) highlight a need to re-engineer the current acute care model towards an ICM [34]. Following the Behavior Change Wheel methodology, we considered the identified determinants to help us choose intervention functions and behavioral change techniques, [33, 122]. As patients and clinicians were open to the use of an eHealth technology, but expressed concerns that technology might replace human contact, the SMILe-ICM intervention includes both human- and technology-based components [33]. The adaptions of the SMILe-ICM and its implementation strategies followed the FRAME and the FRAME-IS framework (Valenta S, Ribaut J, Leppla L, Mielke J, Teynor A, Koehly K, Gerull S, Grossmann F, Witzig-Brändli V, De Geest S, for the SMILe study team: Context-specific adaptation of an eHealth-facilitated, integrated care model and tailoring its implementation strategies – a mixed-methods study as a part of the SMILe implementation science project, Under review). The ERIC taxonomy was used to choose and describe context-specific implementation strategies (e.g., conducting local consensus discussions, creating new clinical teams) [34, 123]. In addition, the contextual analysis itself represented a valuable implementation strategy: conducting a local need assessment. Finally, as part of phase B, the implementation pathway and outcomes (i.e., acceptability, appropriateness, feasibility, and fidelity) will be assessed from a patients’ and healthcare providers’ perspective and likely influences of context considered [32].

### Component 6: Reporting of contextual analysis

As contextual analysis informs subsequent phases of an IS project—affecting, for example, intervention development—it is a critical component of that project and needs to be reported as such [124, 125]. However, given the limited space available in journal articles, detailed findings of contextual analyses and their uses should be reported in separate papers. These are by no means restricted to dedicated IS journals but can also include journals with a clinical focus [126]. Furthermore, a much more serious impediment to the reporting and dissemination of contextual findings is the lack of clear, comprehensive guidelines on how to report contextual analyses [124, 125]. For instance, the Standards for Reporting Implementation Studies (StaRI Checklist) recommend the CFIR for reporting relevant contextual factors; however, information specifying which aspects of the contextual analysis to report is missing [20, 127, 128].

#### Case example — reporting of the SMILe contextual analysis

The SMILe project’s contextual analysis findings for its first study site were published in a separate paper. The same paper described the research team’s implementation strategies and outlined their findings’ implications for re-engineering stem cell transplant follow-up care [34]. A second paper described how the research team had based their intervention component and mode-of-delivery choices on information from their contextual analysis [33]. Applying the BANANA approach to the SMILe project, we were focusing sharply on making our decision-making processes and results transparent and replicable. That is, at each step, we are ensuring that both the results and the processes used to achieve them can be employed by other researchers (e.g., for scale-up).

## Discussion

**Fig. 2:**
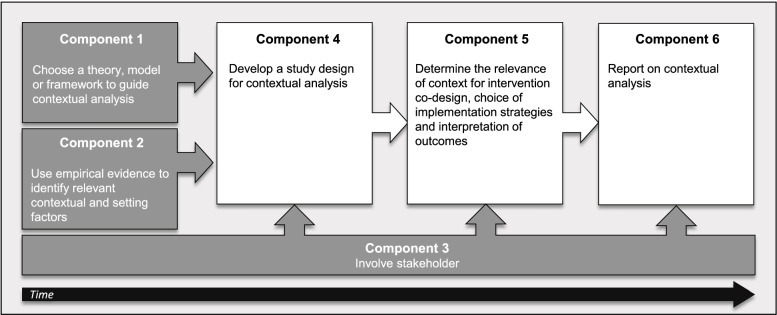
Overview of the six components of the Basel Approach for coNtextual ANAlysis (BANANA)

Contextual analysis should be the foundation of every IS project in our view. As noted above, contextual analysis results inform all subsequent project phases, enhancing interventions’ implementation and sustainability in real-world settings. In comparison to previous studies on facilitators and barriers, the BANANA approach does not only describe individual methods to study context (e.g., surveys or interviews), it provides methodological guidance on planning and conducting contextual analyses in IS projects. Furthermore, BANANA describes how contextual information can be reported and used to inform further project phases (e.g., intervention development). While we have described BANANA in terms of six individual components, neither all components have to be performed in every contextual analysis, nor do they always operate sequentially (Fig. 2). Particularly the first three—choosing a TMF, identifying empirical evidence, and involving stakeholders—are partly concurrent or can be executed in a different order with stakeholder involvement being a key component that is linked to all other components. Once in place, they form a firm foundation upon which to identify and assess relevant contextual factors (component 4).

When presenting BANANA at conferences or in workshops, participants often ask us for a checklist they can apply to their project’s contextual analysis. However, we have deliberately avoided a “checklist approach”: the aspects to be studied in context and the methods chosen always depend on the individual research project and its research questions. Applying a checklist risks oversimplifying the context and undervaluing the complex interconnections of contextual factors, many of which differ from one setting to the next [77, 129]. In the worst cases, only superficial contextual knowledge would be generated, limiting the contextual analyses’ capacity either to inform later phases of the IS project or to ensure the implementation’s success [77, 130]. Therefore, planning and conducting a contextual analysis usually requires a high degree of reflexivity and an experienced transdisciplinary research team covering experiences in the field of IS (e.g., knowledge and use of implementation TMFs, understanding of all implementation phases), and a broad knowledge of how to apply research methods.

In addition to the project and research questions, however, pre-existing contextual knowledge and the researcher’s role influences planning and conducting of a more targeted contextual analysis [38, 131]. The SMILe project leaders (LL, SV) had both worked for several years in the SMILe-ICM’s target setting. For example, both have ample experience in the care of stem cell-transplanted patients as well as implicit and explicit knowledge of the target context and setting (e.g., work processes, resources available, leadership, organizational culture, and legal aspects). These experiences and their role within the clinical setting may have been influential in shaping the focus of our contextual analysis [38]. Given their background as advanced practice nurses, specific values, assumptions, and beliefs (i.e., mental models) drive their understanding of context. This includes, for example, which contextual factors they perceive as relevant to affect implementation, or how they interpret specific findings of the contextual analysis (i.e., confirmation bias). However, the use of a theoretical underpinning to guide contextual analyses (i.e., the eCCM and CICI framework), as well as various evidence sources in addition to the project leader’s professional knowledge might counteract potential bias [38]. Furthermore, by being embedded in the context, they have an insider perspective that may ultimately be helpful not only for conducting the context analysis (e.g., involving stakeholders from the setting), but also to support the implementation and sustainability of the intervention in practice [38, 132].

Second, although the importance of context in IS projects has been widely emphasized, funding agencies remain hesitant to fund contextual analyses as they are not yet recognized as a crucial part of an IS project. A contextual analysis’ rigor and thoroughness both reflect the available resources such as time, personnel, and especially funding [74, 75]. These circumstances should be considered when evaluating contextual analysis and interpreting its results. In projects, where resources are constrained, we do not recommend omitting components of BANANA that a relevant to the individual project, but rather thinking about how the components are carried out. In terms of empirical evidence, for example, informal meetings with key stakeholders could provide sufficient understanding of relevant contextual factors so that the amount of data collected during the contextual analysis can be narrowed down. Other aspects that can be considered in terms of resources include the timeframe of the contextual analysis (e.g., one vs. more timepoints), the number of participants involved, or the methods chosen (e.g., use of rapid qualitative methods). Informal conversations with stakeholders, for example, can also complement data collection or recurrent measures of context, when changes in context need to be observed. In addition, funding agencies need to acknowledge contextual analysis as a foundational phase in IS projects and provide specific funding mechanisms to adequately resource this phase.

### Strengths and limitations

BANANA was developed based on evidence and expert discussion and successfully applied within the SMILe project to guide intervention development [34] as well as intervention adaptation ([35]; Valenta S, Ribaut J, Leppla L, Mielke J, Teynor A, Koehly K, Gerull S, Grossmann F, Witzig-Brändli V, De Geest S, for the SMILe study team: Context-specific adaptation of an eHealth-facilitated, integrated care model and tailoring its implementation strategies – a mixed-methods study as a part of the SMILe implementation science project, Under review). Its six components provide an overall, theory-based guidance for “how to do” contextual analyses in IS projects and raise questions in regard to contextual analysis that needs to be answered individually for each project. BANANA is not limited to the stem cell transplant population or any clinical settings, yet contextual analyses based on the principles of BANANA were already conducted in community-based care [133] and geriatric care [134]. Further application of BANANA for example in the community pharmacy setting is planned. Thereby, based on our operationalization of contextual analysis, BANANA is particularly useful for earlier stage work (e.g., preparatory work, hybrid 1 studies) as well as IS studies that include the development/adaptation, implementation, and evaluation of an intervention and its implementation strategies. For studies focusing on the sustainment or scale-up of interventions the extent to which components of BANANA are relevant might differ. Additional testing will be necessary to ensure its reliability for other project phases (e.g., sustainability, scale-up) and other settings (e.g., in low- and middle-income countries). Furthermore, we are considering methods of finding a broader consensus between implementation experts regarding BANANA’s six components, e.g., by applying a Delphi approach. Another limitation of BANANA is that interactions in context—particularly regarding how individuals are embedded within a context, and how they are influenced by and shape that context—require more consideration than was possible within the scope of this study. Therefore, we plan to further develop BANANA and complement it via social science elements [38].

### Implications for research and funders

Improving researchers’ consideration of context and their reporting of it in IS studies will clearly require conceptual and methodological developments; however, further measures are also required. First, coupled with the acknowledgment of contextual analysis as the foundational first phase of every IS project, its relevance to implementation success requires funding agencies to rethink how to support this phase. That is, adequately resourcing contextual analyses will require specific funding schemes [77]. Within reasonable tolerances, this will require a timeline for a thorough contextual analysis and further components (e.g., intervention development) [77]. Second, the reporting of context should be a condition for the publication of IS projects. Appropriate standards and guidelines must be developed to support researchers to meet this requirement.

## Conclusions

Contextual analysis is a foundational phase of every IS project, providing essential information to all subsequent phases. The BANANA approach successfully guided the SMILe project’s contextual analysis. To help researchers make sense of their target contexts, and to strengthen every part of their work, this approach’s principles can also be applied to other IS projects. However, further adaption and testing of BANANA in other projects are required. Equally importantly, considering the vast heterogeneity of the studies we reviewed, a coordinated campaign will be required to unify and enhance IS researchers’ efforts to conduct and report on contextual analyses. As a first step, a common set of analysis and reporting guidelines will do much to improve the success and quality of implementation efforts.

## Supplementary Information

**Additional file 1.** The Basel Approach for coNtextual ANAlysis (BANANA): Overview of its development process and theoretical underpinning.

**Additional file 2.** Key resources for each component of the Basel Approach for coNtextual ANAlysis (BANANA).

**Additional file 3.** Overview of contextual factors most commonly reported in empirical evidence to influence implementation.

**Additional file 4.** Overview of variables assessed and themes explored in the SMILe project.

## Data Availability

Data analyzed during this study are available from the corresponding author upon reasonable request.

## Abbreviations

BANANA: Basel Approach for coNtextual ANAlysis in implementation science
CICI framework: Context and Implementation of Complex Interventions framework
eCCM: eHealth Enhanced Chronic Care Model
ICM: Integrated care model
IS: Implementation science
TMF: Theory, model, or framework

## Footnotes

1 Context can be defined as “a set of characteristics and circumstances that consist of active and unique factors, within which the implementation is embedded” [1]. Context is multilevel, multidimensional, and dynamic. It interacts with an intervention and its implementation in the setting, i.e., the “physical location, in which an intervention is put into practice” [1].

2 Document analysis can be quantitative as well.

3 Four phases of the MRC guidance: (1) developing a new intervention or identifying an already existing intervention, (2) assessing the feasibility and acceptability of an intervention, (3) assessing an intervention (evaluation), and (4) implementing an intervention [114].
