# Supplementary material for: Unraveling implementation context: the Basel Approach for coNtextual ANAlysis (BANANA) in implementation science and its application in the SMILe project
Source: Implement Sci Commun. 2022 Oct 1;3:102. doi: 10.1186/s43058-022-00354-7 (PMC9526967; doi:10.1186/s43058-022-00354-7)
Supplement: Supplementary file 1 — Additional file 1. The Basel Approach for coNtextual ANAlysis (BANANA): Overview of its development process and theoretical underpinning. [file 43058_2022_354_MOESM1_ESM.docx]

**Additional file 1:** The Basel Approach for coNtextual ANAlysis (BANANA): Overview of its development process and theoretical underpinning

| **Approach Stange and Glasgow** [1] | **Basel Approach for coNtextual ANAlysis (BANANA)^1^** | |
| --- | --- | --- |
|  | **Initial version** | **Final version** |
| Identifying relevant contextual factors based on theory, local history, and the perspectives of multiple stakeholders at the beginning of a project. | Step 1: Choose relevant theories: context/setting (CICI [2] & setting specific theory) | Component 1: Choose a theory, model or framework (TMF) to guide contextual analysis  Considerations when selecting a TMF for contextual analysis   - TMF acknowledges the multidimensional, multilevel and dynamic nature of context - TMF fits the intervention and/or setting in which the intervention will be implemented   *Consider combination of a context and setting specific TMF* |
|  | Step 2: Use available empirical evidence: e.g. barriers/facilitators/practice patterns | Component 2: Use empirical evidence to identify relevant contextual and setting factors  Identification of empirical evidence on relevant contextual and setting factors for  implementation using various sources of evidence   - local data and information - professional knowledge/clinical experience - patient experiences and preferences - research |
|  | Step 3: Stakeholder involvement is essential | Component 3: Involve stakeholder   - Identification and listing of relevant stakeholders for contextual analysis (target group, implementers, decision makers, other) from different levels (micro-, meso-, macro) - Mapping of stakeholders in a stakeholder matrix specifying their characteristics (e.g., influence, role, activity, product) - Visualizing stakeholder characteristics in an influence-interest-capacity matrix - Verifying stakeholder availability and commitment - Developing a stakeholder strategy specifying stakeholder tasks, timepoints and methods for involvement - Evaluation of stakeholder involvement and adaption if needed |
| Collecting and analyzing contextual data at multiple time point during the study | Step 4: Collecting and analyzing data: Mixed methods (QUAN/qual or QUAN/QUAL)  Explore contextual/setting factors & practice patterns using theories and choose appropriate quantitative measures (longitudinal assessment needs to be considered)  Qualitative methods are integral part | Component 4: Develop a study design for contextual analysis  Data collection is guided by theory, empirical evidence and stakeholder input   - Choice of appropriate methods to answer the research questions such as - Quantitative methods (e.g., survey, routine data) - Qualitative methods (e.g., interview, focus group, observation) - (Rapid) ethnography   *Consider changes of context over time*  *Plan (if possible) several timepoints for data collection (e.g., prior, during and at the end of the project)* |
| Reporting relevant contextual factors and how they affected important processes and outcomes. | Step 5: Determine relevance of contextual/setting factors for implementation strategies/outcomes and intervention co-design | Component 5: Determine the relevance of context for intervention co-design, choice of implementation strategies and interpretation of outcomes  Findings from the contextual analysis can be used for:   - Development/adaption of the intervention - Choice/adaption of implementation strategies - Interpretation of implementation and effectiveness outcomes - Choice of sustainability strategies   *Consider development of a program theory to describe/visualize causal pathways between intervention components, implementation strategies and contextual factors* |
|  | Step 6: Publish findings of contextual analysis (can be done as separate paper yet need also to be part of the reporting of implementation science study) | Component 6: Report on contextual analysis  Reporting contextual analysis as part of the implementation intervention study (detailed findings can be reported in a separate paper)  Suggestions for reporting based on BANANA:   - Definition of context and operationalizations of contextual and setting factors studied - TMF applied for contextual and setting analysis and description how it was used - Overview of empirical evidence identified and used - Stakeholder involvement (i.e., stakeholder strategy) - Reporting methods applied for data collection and analysis (e.g., study design, measures used, contextual and setting factors assessed) - Use of findings from the contextual analysis for subsequent project phases (cf. component 5) |
| Note. ^1^ Aspects of BANANA that are conceptually based on the Context and Implementation of Complex Interventions (CICI) framework [2] are highlighted in blue. | | |

**References:**

1. Stange KC, Glasgow RE: **Contextual factors: the importance of considering and reporting on context in research on the patient-centered medical home**. Rockville, MD: Agency for Healthcare Research and Quality; 2013. ARHQ Publication No. 13-0045-EF.

2. Pfadenhauer LM, Gerhardus A, Mozygemba K, Lysdahl KB, Booth A, Hofmann B, Wahlster P, Polus S, Burns J, Brereton L *et al*: **Making sense of complexity in context and implementation: the Context and Implementation of Complex Interventions (CICI) framework**. *Implement Sci* 2017, **12**(1):21.
