# Supplementary material for: Unraveling implementation context: the Basel Approach for coNtextual ANAlysis (BANANA) in implementation science and its application in the SMILe project
Source: Implement Sci Commun. 2022 Oct 1;3:102. doi: 10.1186/s43058-022-00354-7 (PMC9526967; doi:10.1186/s43058-022-00354-7)
Supplement: Supplementary file 2 — Additional file 2. Key resources for each component of the Basel Approach for coNtextual ANAlysis (BANANA). [file 43058_2022_354_MOESM2_ESM.docx]

**Additional file 2:** Key resources for each component of the Basel Approach for coNtextual ANAlysis (BANANA)

| **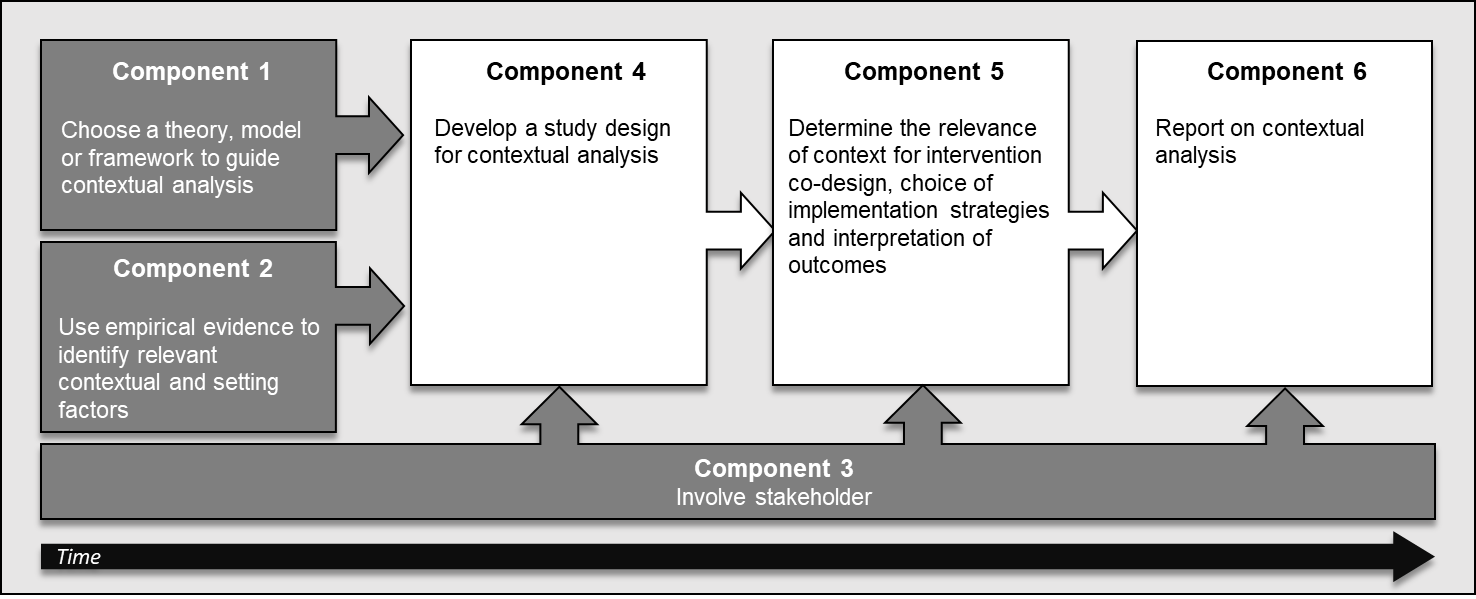** |
| --- |
| Overview of the six components of the Basel Approach for coNtextual ANAlysis (BANANA) |

**Background information**

Papers on context and contextual analysis in general

Squires JE, Graham I, Bashir K, Nadalin-Penno L, Lavis J, Francis J, Curran J, Grimshaw JM, Brehaut J, Ivers N et al: Understanding context: A concept analysis. Journal of Advanced Nursing 2019, 0(0):1-23.

Pfadenhauer LM, Mozygemba K, Gerhardus A, Hofmann B, Booth A, Lysdahl KB, Tummers M, Burns J, Rehfuess EA: Context and implementation: A concept analysis towards conceptual maturity. Zeitschrift fur Evidenz, Fortbildung und Qualitat im Gesundheitswesen 2015, 109(2):103-114.

May CR, Johnson M, Finch T: Implementation, context and complexity. Implementation Science 2016, 11(1):141.

Meier N, Dopson S (eds.): Context in Action and How to Study It: Illustrations from Health Care, 1 edn. United States: Oxford University Press; 2019.

Stange K, Glasgow R: Contextual factors: the importance of considering and reporting on context in research on the patient-centered medical home. Rockville: Agency for Healthcare Research and Quality 2013.

Craig P, Di Ruggiero E, Frolich KL, Mykhalovskiy E, White M, Campbell R, Cummins S, Edwards N, Hunt K, Kee F: Taking account of context in population health intervention research: guidance for producers, users and funders of research. 2018.

Davis M, Beidas RS: Refining contextual inquiry to maximize generalizability and accelerate the implementation process. Implement Res Pract 2021, 2:2633489521994941.

**BANANA – Component 1**

*Use of a theory, model or framework guiding contextual analysis*

***Reviews on TMFs - General***

Tabak RG, Khoong EC, Chambers DA, Brownson RC: Bridging Research and Practice: Models for Dissemination and Implementation Research. American Journal of Preventive Medicine 2012, 43(3):337-350.

Strifler L, Cardoso R, McGowan J, Cogo E, Nincic V, Khan PA, Scott A, Ghassemi M, MacDonald H, Lai Y et al: Scoping review identifies significant number of knowledge translation theories, models, and frameworks with limited use. Journal of clinical epidemiology 2018, 100:92-102.

Moullin JC, Sabater-Hernandez D, Fernandez-Llimos F, Benrimoj SI: A systematic review of implementation frameworks of innovations in healthcare and resulting generic implementation framework. Health Res Policy Syst 2015, 13:16.

Mitchell SA, Fisher CA, Hastings CE, Silverman LB, Wallen GR: A thematic analysis of theoretical models for translational science in nursing: Mapping the field. Nursing Outlook 2010, 58(6):287-300.

Moullin JC, Sabater-Hernandez D, Fernandez-Llimos F, Benrimoj SI: A systematic review of implementation frameworks of innovations in healthcare and resulting generic implementation framework. Health Res Policy Syst 2015, 13:16.

***TMFs including context***

Nilsen P, Bernhardsson S: Context matters in implementation science: a scoping review of determinant frameworks that describe contextual determinants for implementation outcomes. BMC Health Services Research 2019, 19(1):189.

Stange K, Glasgow R: Contextual factors: the importance of considering and reporting on context in research on the patient-centered medical home. Rockville: Agency for Healthcare Research and Quality 2013.

Damschroder LJ, Aron DC, Keith RE, Kirsh SR, Alexander JA, Lowery JC: Fostering implementation of health services research findings into practice: a consolidated framework for advancing implementation science. Implementation Science 2009, 4:50.

Kirk MA, Kelley C, Yankey N, Birken SA, Abadie B, Damschroder L: A systematic review of the use of the consolidated framework for implementation research. Implementation Science 2015, 11(1):72.

Rycroft-Malone J: The PARIHS Framework—A Framework for Guiding the Implementation of Evidence-based Practice. Journal of Nursing Care Quality 2004, 19(4):297-304.

Rycroft-Malone J, Seers K, Chandler J, Hawkes CA, Crichton N, Allen C, Bullock I, Strunin L: The role of evidence, context, and facilitation in an implementation trial: implications for the development of the PARIHS framework. Implementation Science 2013, 8:28.

Harvey G, Kitson A: PARIHS revisited: from heuristic to integrated framework for the successful implementation of knowledge into practice. Implement Sci 2016, 11:33.

Pfadenhauer LM, Gerhardus A, Mozygemba K, Lysdahl KB, Booth A, Hofmann B, Wahlster P, Polus S, Burns J, Brereton L et al: Making sense of complexity in context and implementation: the Context and Implementation of Complex Interventions (CICI) framework. Implementation Science 2017, 12(1):21.

Atkins L, Francis J, Islam R, O’Connor D, Patey A, Ivers N, Foy R, Duncan EM, Colquhoun H, Grimshaw JM et al: A guide to using the Theoretical Domains Framework of behaviour change to investigate implementation problems. Implementation Science 2017, 12(1):77.

***Selecting TMFs***

D&I Models Webtool (<https://dissemination-implementation.org/>)

Birken SA, Powell BJ, Shea CM, Haines ER, Alexis Kirk M, Leeman J, Rohweder C, Damschroder L, Presseau J: Criteria for selecting implementation science theories and frameworks: results from an international survey. Implementation Science 2017, 12(1):124.

Birken SA, Rohweder CL, Powell BJ, Shea CM, Scott J, Leeman J, Grewe ME, Alexis Kirk M, Damschroder L, Aldridge WA et al: T-CaST: an implementation theory comparison and selection tool. Implementation Science 2018, 13(1):143.

Moullin JC, Dickson KS, Stadnick NA, Albers B, Nilsen P, Broder-Fingert S, Mukasa B, Aarons GA: Ten recommendations for using implementation frameworks in research and practice. Implementation Science Communications 2020, 1(1):42.

Minogue V, Matvienko-Sikar K, Hayes C, Morrissey M, Gorman G, Terres A: The usability and applicability of knowledge translation theories, models, and frameworks for research in the context of a national health service. Health Res Policy Syst 2021, 19(1):105.

***Categorizing TMFs***

Nilsen P: Making sense of implementation theories, models and frameworks. Implementation Science 2015, 10(1):53.

**BANANA – Component 2**

*Use of empirical evidence for contextual analysis*

Squires JE, Aloisio LD, Grimshaw JM, Bashir K, Dorrance K, Coughlin M, Hutchinson AM, Francis J, Michie S, Sales A et al: Attributes of context relevant to healthcare professionals’ use of research evidence in clinical practice: a multi-study analysis. Implementation Science 2019, 14(1):52.

Rogers L, De Brún A, McAuliffe E: Defining and assessing context in healthcare implementation studies: a systematic review. BMC Health Serv Res 2020, 20(1):591.

Li S-A, Jeffs L, Barwick M, Stevens B: Organizational contextual features that influence the implementation of evidence-based practices across healthcare settings: a systematic integrative review. Systematic Reviews 2018, 7(1):72.

Watson DP, Adams EL, Shue S, Coates H, McGuire A, Chesher J, Jackson J, Omenka OI: Defining the external implementation context: an integrative systematic literature review. BMC Health Services Research 2018, 18(1):209.

Squires JE, Aloisio LD, Grimshaw JM, Bashir K, Dorrance K, Coughlin M, Hutchinson AM, Francis J, Michie S, Sales A et al: Attributes of context relevant to healthcare professionals’ use of research evidence in clinical practice: a multi-study analysis. Implementation Science 2019, 14(1):52.

**BANANA – Component 3**

*Stakeholder involvement in contextual analysis*

INVOLVE: Briefing notes for researchers: involving the public in NHS, public health and social care research. INVOLVE; 2012. (<https://www.nihr.ac.uk/documents/briefing-notes-for-researchers-public-involvement-in-nhs-health-and-social-care-research/27371>)

PARADIGM Patient Engagement Toolbox (<https://imi-paradigm.eu/>)

Greenhalgh T, Hinton L, Finlay T, Macfarlane A, Fahy N, Clyde B, Chant A: Frameworks for supporting patient and public involvement in research: Systematic review and co-design pilot. Health Expectations 2019, 22(4):785-801.

Gray-Burrows KA, Willis TA, Foy R, Rathfelder M, Bland P, Chin A, Hodgson S, Ibegbuna G, Prestwich G, Samuel K: Role of patient and public involvement in implementation research: a consensus study. BMJ quality & safety 2018, 27(10):858-864.

Barkhordarian A, Demerjian G, Jan A, Sama N, Nguyen M, Du A, Chiappelli F: Stakeholder engagement analysis - a bioethics dilemma in patient-targeted intervention: patients with temporomandibular joint disorders. Journal of Translational Medicine 2015, 13(1):15.

Churruca K, Ludlow K, Taylor N, Long JC, Best S, Braithwaite J: The time has come: Embedded implementation research for health care improvement. Journal of evaluation in clinical practice 2019.

Ramanadhan S, Davis MM, Armstrong R, Baquero B, Ko LK, Leng JC, Salloum RG, Vaughn NA, Brownson RC: Participatory implementation science to increase the impact of evidence-based cancer prevention and control. Cancer Causes & Control 2018, 29(3):363-369.

Piat M, Wainwright M, Sofouli E, Albert H, Casey R, Rivest M-P, Briand C, Kasdorf S, Labonté L, LeBlanc S et al: The CFIR Card Game: a new approach for working with implementation teams to identify challenges and strategies. Implementation Science Communications 2021, 2(1):1.

Bernstein SL, Weiss J, Curry L: Visualizing implementation: contextual and organizational support mapping of stakeholders (Cosmos). Implementation Science Communications 2020, 1:1-11.

**BANANA – Component 4**

*Choosing a study design for contextual analysis*

***Asessing context***

Stange K, Glasgow R: Contextual factors: the importance of considering and reporting on context in research on the patient-centered medical home. Rockville: Agency for Healthcare Research and Quality 2013.

Craig P, Di Ruggiero E, Frolich KL, Mykhalovskiy E, White M, Campbell R, Cummins S, Edwards N, Hunt K, Kee F: Taking account of context in population health intervention research: guidance for producers, users and funders of research. 2018.

Evans JM, Grudniewicz A, Gray CS, Wodchis WP, Carswell P, Baker GR: Organizational context matters: a research toolkit for conducting standardized case studies of integrated care initiatives. International Journal of Integrated Care 2017, 17(2).

Szymczak JE: Beyond barriers and facilitators: the central role of practical knowledge and informal networks in implementing infection prevention interventions. BMJ Quality &amp; Safety 2018, 27(10):763-765.

***Measurement and data extraction tools for specific frameworks***

CFIR (<https://cfirguide.org/>)

EPIS framework (<https://episframework.com/>)

CICI framework appendices; Pfadenhauer LM, Gerhardus A, Mozygemba K, Lysdahl KB, Booth A, Hofmann B, Wahlster P, Polus S, Burns J, Brereton L et al: Making sense of complexity in context and implementation: the Context and Implementation of Complex Interventions (CICI) framework. Implementation Science 2017, 12(1):21.

***Quantitative methods and measures***

Lewis CC, Stanick CF, Martinez RG, Weiner BJ, Kim M, Barwick M, Comtois KA: The Society for Implementation Research Collaboration Instrument Review Project: A methodology to promote rigorous evaluation. Implementation Science 2015, 10(1):2

Chaudoir SR, Dugan AG, Barr CH: Measuring factors affecting implementation of health innovations: a systematic review of structural, organizational, provider, patient, and innovation level measures. Implementation Science 2013, 8:22.

McHugh S, Dorsey CN, Mettert K, Purtle J, Bruns E, Lewis CC: Measures of outer setting constructs for implementation research: A systematic review and analysis of psychometric quality. Implementation Research and Practice 2020, 1:2633489520940022.

Clinton-McHarg T, Yoong SL, Tzelepis F, Regan T, Fielding A, Skelton E, Kingsland M, Ooi JY, Wolfenden L: Psychometric properties of implementation measures for public health and community settings and mapping of constructs against the Consolidated Framework for Implementation Research: a systematic review. Implementation Science 2016, 11(1):148.

Kien C, Schultes M-T, Szelag M, Schoberberger R, Gartlehner G: German language questionnaires for assessing implementation constructs and outcomes of psychosocial and health-related interventions: a systematic review. Implementation Science 2018, 13(1):150.

Gagnon M-P, Attieh R, Ghandour EK, Légaré F, Ouimet M, Estabrooks CA, Grimshaw J: A Systematic Review of Instruments to Assess Organizational Readiness for Knowledge Translation in Health Care. PLOS ONE 2014, 9(12):e114338.

Fernandez ME, Walker TJ, Weiner BJ, Calo WA, Liang S, Risendal B, Friedman DB, Tu SP, Williams RS, Jacobs S et al: Developing measures to assess constructs from the Inner Setting domain of the Consolidated Framework for Implementation Research. Implementation Science 2018, 13(1):52.

Chor KHB, Wisdom JP, Olin S-CS, Hoagwood KE, Horwitz SM: Measures for Predictors of Innovation Adoption. Administration and Policy in Mental Health and Mental Health Services Research 2015, 42(5):545-573.

Estabrooks CA, Squires JE, Cummings GG, Birdsell JM, Norton PG: Development and assessment of the Alberta Context Tool. BMC Health Services Research 2009, 9(1):234.

Rabin BA, Lewis CC, Norton WE, Neta G, Chambers D, Tobin JN, Brownson RC, Glasgow RE: Measurement resources for dissemination and implementation research in health. Implementation Sci 11, 42 (2015). https://doi.org/10.1186/s13012-016-0401-y

Powell BJ, Mettert KD, Dorsey CN, et al. Measures of organizational culture, organizational climate, and implementation climate in behavioral health: A systematic review. Implementation Research and Practice. January 2021. doi:10.1177/26334895211018862

Weiner BJ, Mettert KD, Dorsey CN, Nolen EA, Stanick C, Powell BJ, Lewis CC: Measuring readiness for implementation: A systematic review of measures’ psychometric and pragmatic properties. Implementation Research and Practice 2020, 1:2633489520933896.

***Qualitative methods***

Ramanadhan S, Revette AC, Lee RM, Aveling EL: Pragmatic approaches to analyzing qualitative data for implementation science: an introduction. Implementation Science Communications 2021, 2(1):70.

Howarth E, Devers K, Moore G, O’Cathain A, Dixon-Woods M: Contextual issues and qualitative research. In: Challenges, solutions and future directions in the evaluation of service innovations in health care and public health. vol. 4: Health Serv Deliv Res; 2016: 105-120.

National Cancer Institute: Qualitative Methods in Implementation Science; 2015.

Hamilton AB, Finley EP: Qualitative methods in implementation research: An introduction. Psychiatry Research 2019, 280:112516.

Palinkas LA, Mendon SJ, Hamilton AB: Innovations in Mixed Methods Evaluations. Annual Review of Public Health 2019, 40(1):423-442.

***Mixed-methods***

Palinkas LA, Aarons GA, Horwitz S, Chamberlain P, Hurlburt M, Landsverk J: Mixed Method Designs in Implementation Research. Administration and Policy in Mental Health and Mental Health Services Research 2011, 38(1):44-53.

Palinkas LA: Qualitative and Mixed Methods in Mental Health Services and Implementation Research. Journal of Clinical Child & Adolescent Psychology 2014, 43(6):851-861.

Green CA, Duan N, Gibbons RD, Hoagwood KE, Palinkas LA, Wisdom JP: Approaches to Mixed Methods Dissemination and Implementation Research: Methods, Strengths, Caveats, and Opportunities. Administration and Policy in Mental Health and Mental Health Services Research 2015, 42(5):508-523.

Beidas RS, Wolk CL, Walsh LM, Evans AC, Jr., Hurford MO, Barg FK: A complementary marriage of perspectives: understanding organizational social context using mixed methods. Implementation science : IS 2014, 9:175.

Albright K, Gechter K, Kempe A: Importance of Mixed Methods in Pragmatic Trials and Dissemination and Implementation Research. Academic pediatrics 2013, 13(5):400-407.

Creswell JW, Plano Clark VL: Designing and conducting mixed methods research, 3 edn. Los Angeles: Sage; 2018.

***Rapid methods***

Palinkas LA, Zatzick D: Rapid assessment procedure informed clinical ethnography (RAPICE) in pragmatic clinical trials of mental health services implementation: methods and applied case study. Administration and Policy in Mental Health and Mental Health Services Research 2019, 46(2):255-270.

Nevedal AL, Reardon CM, Opra Widerquist MA, Jackson GL, Cutrona SL, White BS, Damschroder LJ: Rapid versus traditional qualitative analysis using the Consolidated Framework for Implementation Research (CFIR). Implementation Science 2021, 16(1):67.

Vindrola-Padros C, Johnson GA: Rapid Techniques in Qualitative Research: A Critical Review of the Literature. Qualitative Health Research 2020, 30(10):1596-1604.

Vindrola-Padros C, Vindrola-Padros B: Quick and dirty? A systematic review of the use of rapid ethnographies in healthcare organisation and delivery. BMJ Quality &amp; Safety 2018, 27(4):321-330.

***Ethnographic approaches***

Gertner AK, Franklin J, Roth I, Cruden GH, Haley AD, Finley EP, Hamilton AB, Palinkas LA, Powell BJ: A scoping review of the use of ethnographic approaches in implementation research and recommendations for reporting. Implement Res Pract 2021, 2.

Haines ER, Kirk MA, Lux L, Smitherman AB, Powell BJ, Dopp A, Stover AM, Birken SA: Ethnography and user-centered design to inform context-driven implementation. Translational behavioral medicine 2021.

**BANANA – Component 5**

*Identifying and describing relevance of contextual and setting factors for intervention co-design, implementation strategies and outcomes*

***Developing and adapting interventions***

Skivington K, Matthews L, Simpson SA, Craig P, Baird J, Blazeby JM, Boyd KA, Craig N, French DP, McIntosh E et al: A new framework for developing and evaluating complex interventions: update of Medical Research Council guidance. BMJ 2021, 374:n2061.

Bartholomew LK, Parcel GS, Kok G: Intervention Mapping: A Process for Developing Theory and Evidence-Based Health Education Programs. Health Education & Behavior 1998, 25(5):545-563.

O'Cathain A, Croot L, Duncan E, Rousseau N, Sworn K, Turner KM, Yardley L, Hoddinott P: Guidance on how to develop complex interventions to improve health and healthcare. BMJ Open 2019, 9(8):e029954.

Moore G, Campbell M, Copeland L, Craig P, Movsisyan A, Hoddinott P, Littlecott H, O’Cathain A, Pfadenhauer L, Rehfuess E et al: Adapting interventions to new contexts—the ADAPT guidance. BMJ 2021, 374:n1679.

Chambers DA, Norton WE: The Adaptome: Advancing the Science of Intervention Adaptation. American Journal of Preventive Medicine 2016, 51(4, Supplement 2):S124-S131.

Wiltsey Stirman S, Baumann AA, Miller CJ: The FRAME: an expanded framework for reporting adaptations and modifications to evidence-based interventions. Implementation Science 2019, 14(1):58.

Movsisyan A, Arnold L, Copeland L, Evans R, Littlecott H, Moore G, O’Cathain A, Pfadenhauer L, Segrott J, Rehfuess E: Adapting evidence-informed population health interventions for new contexts: a scoping review of current practice. Health Research Policy and Systems 2021, 19(1):13.

Movsisyan A, Arnold L, Evans R, Hallingberg B, Moore G, O’Cathain A, Pfadenhauer LM, Segrott J, Rehfuess E: Adapting evidence-informed complex population health interventions for new contexts: a systematic review of guidance. Implementation Science 2019, 14(1):105.

***Selecting and adapting implementation strategies***

Powell BJ, Waltz TJ, Chinman MJ, Damschroder LJ, Smith JL, Matthieu MM, Proctor EK, Kirchner JE: A refined compilation of implementation strategies: results from the Expert Recommendations for Implementing Change (ERIC) project. Imp Sci 2015, 10:21.

The EPOC taxonomy of health systems interventions. EPOC Resources for review authors. [epoc.cochrane.org/epoc-taxonomy]

EPOC Taxonomy [epoc.cochrane.org/epoc-taxonomy]

Powell BJ, Beidas RS, Lewis CC, Aarons GA, McMillen JC, Proctor EK, Mandell DS: Methods to Improve the Selection and Tailoring of Implementation Strategies. The Journal of Behavioral Health Services & Research 2017, 44(2):177-194.

Waltz TJ, Powell BJ, Fernández ME, Abadie B, Damschroder LJ: Choosing implementation strategies to address contextual barriers: diversity in recommendations and future directions. Implementation Science 2019, 14(1):42.

Miller CJ, Barnett ML, Baumann AA, Gutner CA, Wiltsey-Stirman S: The FRAME-IS: a framework for documenting modifications to implementation strategies in healthcare. Implementation Science 2021, 16(1):36.

Lewis CC, Powell BJ, Brewer SK, Nguyen AM, Schriger SH, Vejnoska SF, Walsh-Bailey C, Aarons GA, Beidas RS, Lyon AR et al: Advancing mechanisms of implementation to accelerate sustainable evidence-based practice integration: protocol for generating a research agenda. BMJ Open 2021, 11(10):e053474.

Lewis CC, Klasnja P, Powell BJ, Lyon AR, Tuzzio L, Jones S, Walsh-Bailey C, Weiner B: From Classification to Causality: Advancing Understanding of Mechanisms of Change in Implementation Science. Frontiers in Public Health 2018, 6(136).

***Influences on implementation and effectiveness outcomes***

Rogers L, De Brún A, Birken Sarah A, Davies C, McAuliffe E: Context counts: a qualitative study exploring the interplay between context and implementation success; 2021.

Chambers DA, Glasgow RE, Stange KC: The dynamic sustainability framework: addressing the paradox of sustainment amid ongoing change. Implementation Science 2013, 8(1):117.

Bruns EJ, Parker EM, Hensley S, Pullmann MD, Benjamin PH, Lyon AR, Hoagwood KE: The role of the outer setting in implementation: associations between state demographic, fiscal, and policy factors and use of evidence-based treatments in mental healthcare. Implementation Science 2019, 14(1):96.

Clavijo-Chamorro MZ, Sanz-Martos S, Gómez-Luque A, Romero-Zarallo G, López-Medina IM: Context as a Facilitator of the Implementation of Evidence-based Nursing: A Meta-synthesis. Western Journal of Nursing Research, 0(0):0193945920914397

Coles E, Anderson J, Maxwell M, Harris FM, Gray NM, Milner G, MacGillivray S: The influence of contextual factors on healthcare quality improvement initiatives: a realist review. Systematic Reviews 2020, 9(1):94.

Meijers JMM, Janssen MAP, Cummings GG, Wallin L, Estabrooks CA, Y.G. Halfens R: Assessing the relationships between contextual factors and research utilization in nursing: systematic literature review. Journal of Advanced Nursing 2006, 55(5):622-635.

Moore GF, Audrey S, Barker M, Bond L, Bonell C, Hardeman W, Moore L, O’Cathain A, Tinati T, Wight D et al: Process evaluation of complex interventions: Medical Research Council guidance. British Medical Journal 2015, 350:h1258.

***Developing a programme theory***

Funnell SC, Rogers PJ. Purposeful program theory: effective use of theories of change and logic models. 1st ed. Jossey-Bass, 2011.

Lawless A, Baum F, Delany-Crowe T, et al. Developing a Framework for a Program Theory-Based Approach to Evaluating Policy Processes and Outcomes: Health in All Policies in South Australia. Int J Health Policy Manag 2018;7:510-21.

**BANANA – Component 6**

*Reporting of contextual analysis*

Pinnock H, Barwick M, Carpenter CR, Eldridge S, Grandes G, Griffiths CJ, Rycroft-Malone J, Meissner P, Murray E, Patel A: Standards for reporting implementation studies (StaRI) statement. bmj 2017, 356:i6795.

Pinnock H, Barwick M, Carpenter CR for the StaRI Group et al., Standards for Reporting Implementation Studies (StaRI): explanation and elaboration document BMJ Open 2017;7:e013318. doi: 10.1136/bmjopen-2016-013318

Wells M, Williams B, Treweek S, Coyle J, Taylor J: Intervention description is not enough: evidence from an in-depth multiple case study on the untold role and impact of context in randomised controlled trials of seven complex interventions. Trials 2012, 13(1):95.

Bertram R, Edwards D, Engell T, Kerns SEU, Øvretveit J, Rojas-Andrade R, Sarkies M, Williams CR: Welcome to Global Implementation Research and Applications. Global Implementation Research and Applications 2021, 1(1):1-4.

Wensing M, Sales A, Wilson P, Armstrong R, Kislov R, Rankin NM, Ramaswamy R, Xu D: Implementation Science and Implementation Science Communications: a refreshed description of the journals’ scope and expectations. Implementation Science 2021, 16(1):103.

Mielke J, Brunkert T, Zullig LL, Bosworth HB, Deschodt M, Simon M, De Geest S: Relevant Journals for Identifying Implementation Science Articles: Results of an International Implementation Science Expert Survey. Frontiers in Public Health 2021, 9(458).
