# Supplementary material for: Unraveling implementation context: the Basel Approach for coNtextual ANAlysis (BANANA) in implementation science and its application in the SMILe project
Source: Implement Sci Commun. 2022 Oct 1;3:102. doi: 10.1186/s43058-022-00354-7 (PMC9526967; doi:10.1186/s43058-022-00354-7)
Supplement: Supplementary file 3 — Additional file 3. Overview of contextual factors most commonly reported in empirical evidence to influence implementation. [file 43058_2022_354_MOESM3_ESM.docx]

**Additional file 3**

Overview of contextual factors most commonly reported in empirical evidence to influence implementation

|  | **Rogers et al. (2020) [1]** | **Squires et al. (2019) [9]** | **Watson et al. (2018) [13]** | **Li et al. (2018) [50]** |
| --- | --- | --- | --- | --- |
| **Micro-level** | Self-efficacy | - | - | - |
|  | Individual attitudes | - | - | - |
|  | - | Patient characteristics | - | - |
|  | - | Health care professional characteristics | - | - |
| **Meso-level** | Culture | Culture | - | Organizational culture |
|  | Organizational climate | - | - | - |
|  | Networks and communications | - | - | Networks and communication |
|  | Organizational leadership engagement | Leadership | - | Leadership |
|  | Available resources | Resource access | - | Resources |
|  | Structural characteristics | Facility characteristics | - | - |
|  | - | Evaluation | - | Evaluation, monitoring and feedback |
|  | - | - | - | Champion |
|  | Compatibility | - | - | - |
|  | Organizational support | - | - | - |
|  | - | System features | - | - |
| **Macro-level** | - | Professional role | Professional influences | - |
|  | Political environment | - | Political support | - |
|  | Social environment | Societal influences | Social climate | - |
|  | - | - | Local infrastructure | - |
|  | - | Regulatory or legislative standards | Policy and legal climate | - |
|  | - | Collaboration | Relational climate | - |
|  | - | - | Target population | - |
|  | Economic environment | Financial | Economic and funding climate | - |
| **Team level** | Structural characteristics | Work structure | - | - |
|  | Teamwork | - | - | - |
|  | Culture | - | - | - |
|  | Compatibility | - | - | - |
|  | Available resources | - | - | - |
|  | Local leadership engagement | - | - | - |
|  | Team efficacy | - | - | - |
