# Supplementary material for: Unraveling implementation context: the Basel Approach for coNtextual ANAlysis (BANANA) in implementation science and its application in the SMILe project
Source: Implement Sci Commun. 2022 Oct 1;3:102. doi: 10.1186/s43058-022-00354-7 (PMC9526967; doi:10.1186/s43058-022-00354-7)
Supplement: Supplementary file 4 — Additional file 4. Overview of variables assessed and themes explored in the SMILe project. [file 43058_2022_354_MOESM4_ESM.docx]

**Additional file 4:** Overview of variables assessed and themes explored in the SMILe project

| **Table 1.**  Overview of variables assessed using questionnaire surveys (transplant director`s, clinician`s and patient`s perspective) [1-4] | | |
| --- | --- | --- |
| **Variable** | **Description** | **Measurement** |
| **Structural characteristics alloSCT^1^ center** | *Transplant director`s perspective* 5 items assessing type of alloSCT center, location of alloSCT center, years since start of alloSCT program, number of patients followed up 1-year after alloSCT, alloSCT center size, multidisciplinarity of alloSCT team | Adapted BRIGHT transplant director questionnaire [2] |
| **Practice patterns** | *Transplant director`s perspective* (8 items): length of hospital stay after alloSCT, number of yearly visits after alloSCT, formal mental health or psychological evaluation before alloSCT, formal financial-social evaluation before alloSCT, follow-up in outpatient clinic by same physician, initial contact for after hour questions/emergencies | Adapted BRIGHT transplant director questionnaire [2] |
|  | *Clinician`s perspective* (3 items): having a long-term care-coordinator in the alloSCT clinic, having an Advanced Practice Nurse with specialization in alloSCT, average time per patient in minutes during follow-up | BRIGHT clinician Questionnaire [2] |
|  | *Patient`s perspective* (1 item): time spent with the alloSCT team during follow-up | Self-report (written) questionnaire [2] |
| **Level of chronic illness management** | *Clinicians`s perspective:* 55 items assessing self-management support, delivery system design, clinical decision support, clinical information system | CIMI-BRIGHT [1, 2] |
|  | *Patient`s perspective:* 11 items assessing self-management support, delivery system design, clinical decision support | Short version of the Patient Assessment of Chronic Illness Care (PACIC) instrument [2, 5] |
| **Self-management and health behavior** | *Patient`s perspective:* 52 items assessing level of physical activity, smoking status, alcohol consumption, sun protection, diet adherence, barriers of taking immunosuppressive medication as prescribed, importance and confidence of taking immunosuppressive medication, adherence to appointment keeping, being a member of a patient organization, health literacy | Brief Physical Activity Assessment tool [6], Swiss Health survey [7], adapted BRIGHT patient self-report [2], Swiss study on health of people with cancer, leukemia, tumor in childhood [8] and Cambridge University Hospitals' perception of skin cancer in transplant recipients scale [9] |
| **Self-management support** | *Patient`s perspective* (7 items): advice by alloSCT team on physical activity, alcohol intake, sun protection, diet, intake of immunosuppressive medication; frequency of having someone`s help reading health related material, problems in understanding health related information | Adapted BRIGHT patient self-report [2] |
| **Technology openness** | *Patient`s perspective:* 16 items assessing patient`s technology openness towards eHealth, eHealth experience and usage patterns, overall willingness to use an eHealth device | Investigator developed questionnaire, Adapted PICASSO TX Questionnaire patient self-report [10, 11] |
| **eHealth system in the alloSCT center^2^** | *Transplant director`s perspective:* 4 items assessing eHealth system application in the alloSCT center | Investigator developed [3] |
| **Information on implementation aspects^2^** | *Transplant director`s perspective:* 6 items assessing perceptions of implementing an eHealth supported integrated care model in alloSCT follow-up | Investigator developed [3] |
| **Demographic characteristics** | *Clinicians:* age, gender, position, work experience |  |
|  | *Patients:* age, gender, marital status, educational level, employment status, disability pension |  |

Note. ^1^ alloSCT = allogeneic hematopoietic stem cell transplanted; ^2^ variables were only assessed in the Swiss setting [4]

| **Table 2.** Overview of themes explored in individual interviews and focus group interviews [3, 4] | | |
| --- | --- | --- |
| **Participants** | **Theme/Questions** | **Method** |
| **Clinicians** | *Demographics:* age, gender, profession, work experience with alloSCT^1^ patients | Focus group interviews |
|  | *Follow-up care:* Perceptions of current alloSCT follow-up care and aspects that could be improved and how. Perceptions of frequent avoidable complications for rehospitalization. |  |
|  | *Patient`s self-management:* Perceptions on relevant self-management task and potential barriers to it. Perceptions on relevant information patients should receive to be prepare for the home setting. |  |
|  | *Technology^2^ use in follow-up care:* Perceptions how technology could improve follow-up-care and aspects that would be important to clinicians. Perceptions about barriers to technology use. Perceptions of important features and relevant parameters that could be monitored using technology. |  |
|  | *SMILe implementation^3^:* Perceptions on what it takes to implement SMILe and facilitating or hindering factors to implementation. |  |
| **Patients** | *Demographics:* age, gender, month and year of transplantation, employed (yes/no), relationship (yes/no), number of comorbidities | Focus group interviews^5^ and individual interviews |
|  | *Follow-up care:* Description of current alloSCT follow-up care^4^ and involvement of family care givers in follow-up care. Perceptions of important and stressful aspects in regard to follow-up care. Perceptions of aspects in follow-up care that could be improved and how. |  |
|  | *Patient`s self-management:* Description of tasks/activities performed regularly to maintain/improve the health status. Perceptions of possible barriers to self-management tasks and aspects patients felt unprepared for in the home-setting. |  |
|  | *Technology^2^ use in follow-up care:* Perceptions on how technology could assist patients in follow-up care. Perceptions on aspects that would be important to patients and hindering factors for technology use. |  |

Note. ^1^ alloSCT = allogeneic hematopoietic stem cell transplanted; ^2^ technology refers to internet-based application or app; ^3^ theme was only explored in the Swiss setting [4]; ^4^ e.g., contacts or support received from the hospital, visits to healthcare providers, frequency, scheduling of appointments, transportation, waiting times, financial burden; ^5^ only conducted in the German setting [3]

**References**

1. Berben L, Russell CL, Engberg S, Dobbels F, De Geest S: **Development, content validity and inter-rater reliability testing of the Chronic Illness Management Implementation – Building Research Initiative Group: Chronic Illness Management and Adherence in Transplantation: An instrument to assess the level of chronic illness management implemented in solid organ transplant programmes**. *Int J Care Coord* 2014, **17**(1-2):59-71.

2. Berben L, Denhaerynck K, Dobbels F, Engberg S, Vanhaecke J, Crespo-Leiro MG, Russell CL, De Geest S, consortium tBs: **Building research initiative group: chronic illness management and adherence in transplantation (BRIGHT) study: study protocol**. *J Adv Nurs* 2015, **71**(3):642-654.

3. Leppla L, Mielke J, Kunze M, Mauthner O, Teynor A, Valenta S, Vanhoof J, Dobbels F, Berben L, Zeiser R *et al*: **Clinicians and patients perspectives on follow-up care and eHealth support after allogeneic hematopoietic stem cell transplantation: A mixed-methods contextual analysis as part of the SMILe study**. *European Journal of Oncology Nursing* 2020, **45**:101723.

4. Valenta S, Ribaut J, Leppla L, Mielke J, Teynor A, Koehly K, Gerull S, Grossmann F, Witzig-Brändli V, De Geest S *et al*: **Context-specific adaptation of an eHealth-facilitated, integrated care model and tailoring its implementation strategies – a mixed-methods study as a part of the SMILe implementation science project**. *Under review*.

5. Gugiu PC, Coryn C, Clark R, Kuehn A: **Development and evaluation of the short version of the Patient Assessment of Chronic Illness Care instrument**. *Chronic Illn* 2009, **5**(4):268-276.

6. Marshall AL, Smith BJ, Bauman AE, Kaur S: **Reliability and validity of a brief physical activity assessment for use by family doctors**. *Br J Sports Med* 2005, **39**(5):294-297.

7. Swiss Federal Statistical Office: **Gesundheit und Gesundheitsverhalten in der Schweiz 2007: Schweizerische Gesundheitsbefragung**. In*.* Neuchâtel, Switzerland: Swiss Federal Statistical Office; 2008.

8. Swiss Childhoo Cancer Registry: **Swiss study on the health of people with cancer, leukemia or tumor in childhood**. In*.*

9. Hussain SH, Metthewes G, Todd P, Chaudhry A: **Skin cancer in renal transplant patients**. In*.*; 2011.

10. Vanhoof JMM, Vandenberghe B, Geerts D, Philippaerts P, De Mazière P, DeVito Dabbs A, De Geest S, Dobbels F, Consortium obotP-T: **Technology Experience of Solid Organ Transplant Patients and Their Overall Willingness to Use Interactive Health Technology**. *J Nurs Scholarsh* 2018, **50**(2):151-162.

11. Vanhoof JM, Vandenberghe B, Geerts D, Philippaerts P, De Mazière P, DeVito Dabbs A, De Geest S, Dobbels F, consortium PT, Dupont L: **Shedding light on an unknown reality in solid organ transplant patients’ self‐management: a contextual inquiry study**. *Clinical transplantation* 2018, **32**(8):e13314.
